# Antibacterial and Hemolytic Activity of Antimicrobial Hydrogels Utilizing Immobilized Antimicrobial Peptides

**DOI:** 10.3390/ijms25084200

**Published:** 2024-04-10

**Authors:** Edvin Blomstrand, Elin Posch, Annija Stepulane, Anand K. Rajasekharan, Martin Andersson

**Affiliations:** 1 Department of Chemistry and Chemical Engineering, Chalmers University of Technology, Kemigården 4, SE-412 96 Göteborg, Sweden; edvin.blomstrand@amferia.com (E.B.); elinpo@chalmers.se (E.P.); annijas@chalmers.se (A.S.); 2 Amferia AB, Astra Zeneca BioVentureHub c/o Astra Zeneca, Pepparedsleden 1, SE-431 83 Mölndal, Sweden; anandk@amferia.com; 3 Centre for Antibiotic Resistance Research in Gothenburg (CARe), SE-405 30 Gothenburg, Sweden

**Keywords:** antimicrobial peptides, hydrogels, antibacterial functionalization, surface attachment, biomaterials

## Abstract

Antimicrobial peptides (AMPs) are viewed as potential compounds for the treatment of bacterial infections. Nevertheless, the successful translation of AMPs into clinical applications has been impeded primarily due to their low stability in biological environments and potential toxicological concerns at higher concentrations. The covalent attachment of AMPs to a material’s surface has been sought to improve their stability. However, it is still an open question what is required to best perform such an attachment and the role of the support. In this work, six different AMPs were covalently attached to a long-ranged ordered amphiphilic hydrogel, with their antibacterial efficacy evaluated and compared to their performance when free in solution. Among the tested AMPs were four different versions of synthetic end-tagged AMPs where the sequence was altered to change the cationic residue as well as to vary the degree of hydrophobicity. Two previously well-studied AMPs, Piscidin 1 and Omiganan, were also included as comparisons. The antibacterial efficacy against *Staphylococcus aureus* remained largely consistent between free AMPs and those attached to surfaces. However, the activity pattern against *Pseudomonas aeruginosa* on hydrogel surfaces displayed a marked contrast to that observed in the solution. Additionally, all the AMPs showed varying degrees of hemolytic activity when in solution. This activity was entirely diminished, and all the AMPs were non-hemolytic when attached to the hydrogels.

## 1. Introduction

Bacteria are often fond of surfaces of both organic and inorganic origin. When the surface in question involves human body tissues or medical devices, like implants, it can potentially lead to significant concerns related to infections. When bacteria are colonizing a surface, many of them have the propensity to form biofilms. Biofilms are communities of bacteria, usually consisting of several different strains, that reside inside a slimy extracellular matrix produced by the bacteria themselves. The biofilm provides the bacteria with protection against host defense and antibiotics and gives them a base to communicate and even exchange genetic material [1]. Furthermore, bacteria residing within a biofilm typically display a different phenotype compared to planktonic bacteria, exhibiting varied metabolic patterns depending on their location within the biofilm, which significantly contributes to their enhanced antimicrobial resistance [2]. As a consequence, if a biofilm is established on an implanted medical device, treatment options are very limited, often resulting in device removal [3]. If a biofilm is formed in a wound, the recommended treatment is to first debride the wound by trying to cut and scrape off the infected tissue followed by using antibacterial substances [4].

It is clear that proactive measures are essential to inhibit bacterial colonization on surfaces integral to our bodies and on materials specifically engineered for interaction with our tissues, where there is a risk of infection. In addition, as the prevalence of antibiotic resistance in bacteria continues to grow and evolve, alternatives to conventional antibiotics are needed. Antimicrobial peptides (AMPs) have been deemed as a promising alternative, on which there has been a steadily growing body of research performed through the last few decades [5]. AMPs are generally amphiphilic with an overall cationic net charge. This makes them membrane-active, which is also how most AMPs are believed to exert their antimicrobial effect [6,7]. While it is challenging to study in detail, a few experimental models have shown that certain AMPs cause lipid disruption and destabilization as well as create pores through lipid membranes [8,9,10]. This disrupts the intracellular and extracellular balance, effectively killing the bacteria. However, this is only one suggested mode of action, where in reality the same peptide could exert its antibacterial effect through several paths and targets [11,12]. This non-specific targeting provides most AMPs with broad-spectrum activity, which in combination with the fact that several components of the bacteria can be affected has also been hypothesized to reduce the risks of resistance [13,14]. Furthermore, both Gram-negative and Gram-positive bacteria as well as some fungi and enveloped viruses have a negatively charged surface, unlike the net neutral charge found on most mammalian cells. This means that a higher affinity is found towards microbes, and AMPs can effectively kill them while remaining harmless to human cells. There are; however, some AMPs that have been linked to exerting their antibacterial effect by targeting and disrupting internal cell components. This internal targeting has, on the other hand, been hypothesized to be more susceptible to resistance development [7].

AMPs are part of the innate immune system of multicellular organisms, including humans, and have therefore been around for millennia [15]. Both naturally derived AMPs and completely synthetically made versions have been used and proven effective against a wide variety of microorganisms, and there are a range of AMPs under investigation for clinical use [14]. However, one major drawback of the adaptation of AMPs into medicine is that they are often sensitive to plasma. Their antimicrobial effect can be inhibited by high ionic strength and interactions with plasma components, and they can also be degraded by enzymatic proteolysis. Raising the AMP dosage to deal with this might lead to better antimicrobial effect—but at the cost of increased cytotoxicity. In order to successfully introduce AMPs into medicine, alternative approaches therefore need to be implemented. Some non-naturally occurring amino acids are, for instance, less sensitive to higher ionic strength and proteolysis, and the inclusion of such acids in AMPs has been shown to be effective [16]. Another strategy to diminish sensitivity involves the use of D-amino acids, which are much less prevalent in nature, and typically exhibit lower proteolysis [17,18]. End capping and cyclization also have an effect on proteolytic stability but might also affect antimicrobial potency [19]. In addition, other researchers have looked into the drug-delivery of AMPs, protecting them from serum until they are released [20,21].

In this present study, the strategy has been to covalently attach AMPs to a surface. This has previously been shown to protect the AMPs while also resulting in a contact-killing antimicrobial material [22]. Depending on the material characteristics they can be of interest in a range of applications. The material used in this study is a soft amphiphilic hydrogel, which may potentially find applications in areas such as wound dressings. The hydrogel consists of a block copolymer, Pluronic F-127, which self-assembles into micelles or lyotropic liquid crystals depending on its concentration in water. In this study, we used a concentration corresponding to the micellar cubic phase [23]. The polymer has been chemically modified with acrylate end groups which allows for cross-linking as well as formation of carboxylic acid groups. These groups are then utilized to form amide bonds with primary amine groups found on the AMPs by using 1-ethyl-3-(3-dimethylaminopropyl) carbodiimide (EDC) and N-hydroxysuccinimide (NHS) coupling. However, by covalently attaching an AMP to a surface there are considerable limitations regarding the degrees of freedom and depending on the mode of action, there is a risk that they will lose their antimicrobial efficacy. For example, if the AMPs manifest their antibacterial impact through pore formation, it is plausible to consider that it becomes considerably more challenging for AMPs to align with each other when they are physically immobilized. Moreover, if the AMP is targeting intracellular components, achieving access to these elements would pose a significant challenge when covalently attached to a material. To circumvent the negative effects of the surface, there are studies showing that having a flexible spacer between a solid material and the AMP significantly increases the antibacterial effect [24,25]. On this note, using a soft hydrogel formed by flexible polymers might be beneficial in that the AMPs are held in place but still retain some mobility. However, it is entirely plausible that a specific AMP may operate via multiple mechanisms, and its function can differ significantly when freely dissolved in solution as opposed to when it is attached to a surface.

The AMPs used in this present study display a few different sets of properties that are of interest to investigate regarding how they perform covalently attached to the soft hydrogel surface. Six different AMPs were investigated in the study, as presented in Table 1. One AMP that our research group has previously studied in detail, with the amino acid sequence RRPRPRPRPWWWW-NH_2_, was included as well as two alterations where arginine was exchanged for lysin and one where an extra tryptophan group was added to the end tagging. The lysin includes a primary amine group as part of the side group, which is used for the EDC/NHS attachment [26]. The hypothesis is that the lysin addition may enable more attachment to the hydrogel than the guanidine group of the arginine if the available amine sites are a limiting factor. One AMP has the two arginines closest to the N-terminal changed to lysine while the other has all arginine changed to lysine. The AMP with the two lysine close to the N-terminal should in theory promote attachment allowing most of the peptide to stick out, while the AMP with all arginine changed to lysine should be more randomly attached. Therefore, by modifying the peptide sequences, it could be determined if their orientation significantly influences antibacterial activity and whether noticeable variations in efficacy can be observed. On the other hand, studies have shown that AMPs containing arginine tend to have a stronger antibacterial effect compared to lysin [27,28]. On another note, substituting lysin and arginine in AMPs with the weaker basic amino acid histidine has actually been shown to lose their antibacterial effect completely in natural pH, only regaining it at acidic pH [29]. 

The second modification of the additional tryptophan is of interest as it increases the hydrophobicity of the AMP. This should result in a more antimicrobial-active AMP but potentially at the cost of being more toxic [30,31]. Omiganan and Piscidin 1 were also included, as they have a wide and potent antibacterial effect and a more diverse amino acid sequence compared to the other end-tagged synthetic AMPs. Furthermore, Piscidin 1, known for adopting an alpha-helical secondary structure, is presumed to be important for expressing its antibacterial properties. This is potentially achieved via the toroidal pore model, which presents an intriguing subject of study within this context [32]. A suggested mechanism of action for Omiganan is membrane destabilization after saturation of the membrane, causing leakage [33]. Hydrophobically end-tagged peptides have been shown to cause cell wall rupture and liposome leakage, but the exact mechanism of achieving this is unknown [30,34]. Piscidin 1 is found in mast cells of fish, with striped bass being the most studied case, and Omiganan is a synthetic analog to Indolicidin which is expressed by bovine neutrophils [35,36].

The aim of this study was to examine the behavior of the aforementioned six AMPs when covalently attached to a soft amphiphilic hydrogel. The objective is to gain deeper insights into the factors that influence the attachment of an AMP and identify the features critical for eliciting an antibacterial effect when attached to the surface as compared to when they are free in solution. Furthermore, we aim to understand how this attachment influences cytotoxicity by performing hemolysis studies.

## 2. Results

Information about the structure, charge and hydrophobicity of the six different peptides used in the study can be found in Table 1. All peptides have a cationic charge between +4 and +6 at physiological pH. The synthetic peptides K9W4, KR9W4, R9W4, and R9W5 are amphiphilic due to a separate hydrophilic domain with either arginine and proline or lysine and proline and then an end-tagged hydrophobic domain consisting of a block of tryptophans. For Omiganan and Piscidin 1, the hydrophobic and hydrophilic amino acids are distributed evenly in the respective sequences, likely due to the presence of a secondary structure. Notably, when interacting with membranes, Piscidin 1 is known to assume an alpha-helical structure, whereby the hydrophilic and hydrophobic sections align to optimize its interaction with the lipid bilayer. The hydrophobicity was calculated based on the Wimley White experimentally determined scale measuring the free energy of transfer from bilayer to water (higher values indicate a more hydrophobic sequence) [37].

The amount of AMP taken up by the hydrogels during the functionalization of hydrogel discs with a diameter of 14 mm and a thickness of 1 mm was determined by UV-vis measurements and is presented in Table 2. K9W4 had the lowest attachment with an average of 119 nmol per hydrogel disc and R9W5 had the highest attachment with 166 nmol per disc. The surface ζ-potential at pH 7.4 is also presented in Table 2. The control hydrogel without any AMP was the only surface that had a negative ζ-potential, while the positive ζ-potential varied for the AMP functionalized hydrogel surfaces. R9W5 had the lowest ζ-potential at 0.09 mV and R9W4 had the highest ζ-potential at 1.41 mV. The water contact angles of the surfaces are also presented in Table 2. The contact angle of the control hydrogel was measured to be 95.5° and the AMP-functionalized surfaces varied from 86.7° for R9W4 to 99.6° for KR9W4.

The minimum inhibitory concentrations (MIC) observed for the different AMPs free in solution against *S. aureus* (CCUG 10778) and *P. aeruginosa* (CCUG 56489) are presented in Table 3. The tests were performed in triplicate, and when variances in MIC values were noted between iterations, a range was presented instead. For *S. aureus*, Piscidin 1 showed the lowest MIC values at 1–2 µm, followed by Omiganan and R9W4. K9W4 had a relatively high MIC at 64–128 µm and KR9W4 and R9W5 were in the middle at 8–16 µm. For *P. aeruginosa*, Piscidin 1 again showed the lowest MIC values at 8–16 µm followed by R9W4 at 16 µm. The other AMPs showed MIC at around 32 µm or higher.

**Figure 1:**
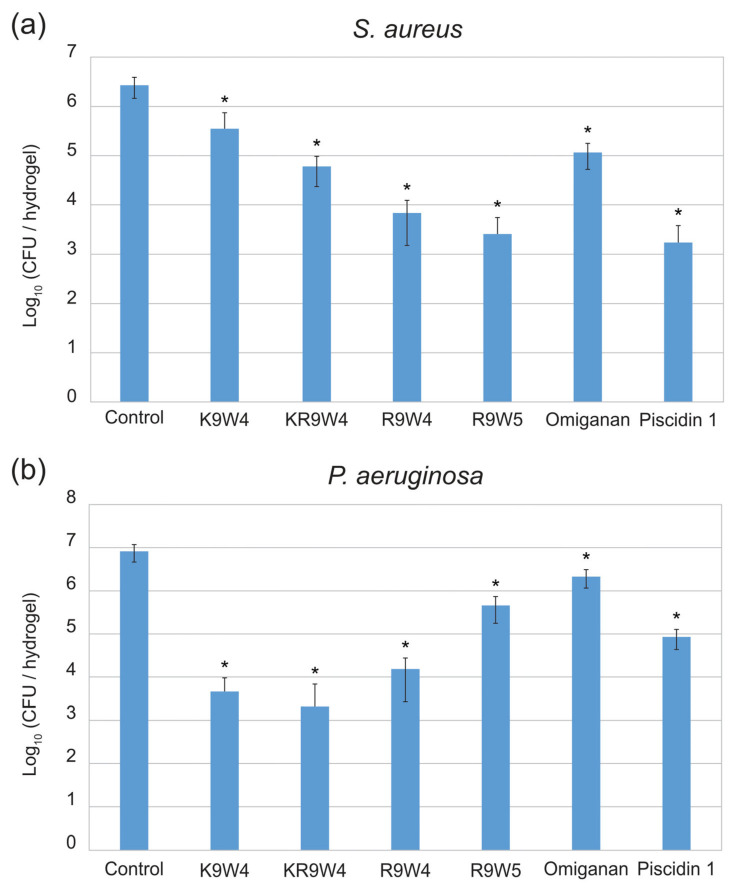
Colony forming units (CFU) observed on the hydrogel surfaces functionalized with six different AMPs as well as a control hydrogel surface. In (**a**) the CFU of *S. aureus* is presented and in (**b**) the CFU of *P. aeruginosa* is presented. The data presented are the average of three runs performed in triplicates (*n* = 9) and is shown in log_10_. The error bars show the standard deviation and * indicates a statistically significant reduction compared to the control, *p* < 0.05.

Results from the antibacterial study performed on the surfaces of hydrogel discs functionalized with the different AMPs are presented in Figure 1. In Figure 1a the colony-forming unit (CFU) of *S. aureus* found for the different surfaces after 30 min of contact time is presented and in Figure 1b the CFU of *P. aeruginosa* found on the different surfaces after 30 min of contact time is presented. The hydrogels functionalized with Piscidin 1 and R9W4 showed the strongest antibacterial effect against *S. aureus* with roughly a 3-log reduction in bacteria present with respect to the AMP-free control hydrogel. The hydrogels functionalized with K9W4 and Omiganan showed the weakest antibacterial activity with roughly 1 log reduction observed against *S. aureus*. For *P. aeruginosa*, the hydrogels functionalized with K9W4 and KR9W4 showed the strongest antibacterial activity with a 3–4 log reduction in bacteria compared to the control. Omiganan functionalized hydrogels showed the weakest antibacterial effect against *P. aeruginosa* with less than 1 log bacterial reduction. There was a statistically significant reduction compared to the control for all samples with a confidence interval of 95%. Furthermore, the different AMP functionalized hydrogels showed a statistically significant (confidence interval of 95%) difference to all other AMP functionalized hydrogels within the same bacteria except for hydrogels functionalized with R9W5 and Piscidin 1 for *S. aureus* which were not statistically different.

**Figure 2:**
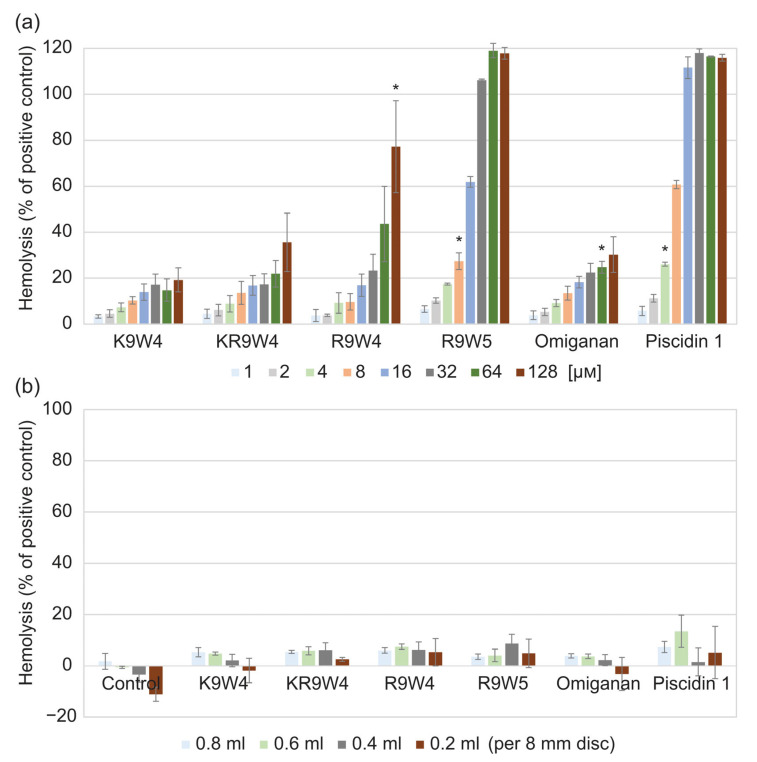
Hemolysis of 1 vol% defibrinated horse blood compared to the positive control (0.1% Triton X-100). (**a**) hemolysis from the free AMPs at different concentrations ranging from 1 µm to 128 µm. Each bar is the average of three runs and the error bars are the standard deviation. (**b**) hemolysis of control hydrogels without any AMPs (noted as control in graph), and hydrogels functionalized by the six different AMPs. The hydrogel discs of the same size were placed in different volumes of the blood solution, 0.2 mL to 0.8 mL. Each bar is the average of three runs performed in duplicates, *n* = 6. * indicates the concentration in which the hemolysis was statistically significantly higher than the set threshold at 20% hemolysis, *p* < 0.05.

Results from the hemolysis study of the AMPs when free in solution are shown in Figure 2a, and results from when they are attached to the hydrogel surface are shown in Figure 2b. While free in solution, the AMP K9W4 did not cross 20% hemolysis for any of the concentrations evaluated. While KR9W4 crossed 20% hemolysis at 64 µm, no concentration was statistically significantly higher. For R9W4, the 20% hemolysis threshold was passed with statistical significance at 128 µm. For Omiganan, the 20% hemolysis threshold was passed with statistical significance at 64 µm. R9W5 crossed the 20% hemolysis threshold at 8 µm and 100% hemolysis was observed at 32 µm. Piscidin 1 showed the strongest hemolytic response as it passed the 20% hemolysis at 4 µm and 100% hemolysis was reached at 16 µm. For the AMP functionalized hydrogels, Figure 2b, there was no strong hemolysis response observed for any of the AMP functionalized hydrogels as the hemolysis levels never rose above the 20% hemolysis threshold.

## 3. Discussion

The results from experiments performed on the AMPs dissolved in solution were largely in line with earlier observations and theoretical predictions as mentioned in the introduction. In contrast, the results from the AMPs attached to the hydrogels were less intuitive. For example, for the AMP attachment, as evaluated by UV-vis, it was clear that changing the arginine amino acids to lysin did not have the hypothesized effect of more AMPs attaching to the hydrogel. Instead, changing all the arginine to lysin resulted in less AMP attachment. On the other hand, this does strongly indicate that the availability of primary amine groups on the peptide is not a limiting factor for the EDC/NHS attachment mechanism. From the data, it instead appears to be the hydrophobicity of the peptides that could be a driving force to increase the peptide functionalization as R9W5 and Omiganan, the two more hydrophobic peptides, showed the highest amount of attachment. An increase in hydrophobicity is expected to yield higher surface activity, thereby creating a stronger impetus for the molecules to migrate from the aqueous solution toward the surfaces. This expectation is further reasonable given that the substrate polymer also contains repeating hydrophobic domains, thereby fostering favorable interactions with more hydrophobic AMPs. Different attachment profiles of AMPs for other substrates with different surface chemistries would therefore not be surprising. The reason why there are no attachment values presented for Piscidin 1 is that it does not contain any tryptophan or tyrosine groups from which the absorbance can be measured. While it does contain phenylalanine which also contains an aromatic ring, the signal from this was not distinguishable from the background.

From the ζ-potential presented in Table 2, it was evident that the AMP functionalization increased the surface charge to positive values as the ζ-potential changed from −1.09 mV for the control hydrogel to positive values for the AMP hydrogel samples. The majority of the AMP functionalized hydrogels showed values in the range 0.6–0.8 mV; however, R9W5 only showed 0.09 mV, while R9W4 showed a higher ζ-potential at 1.42 mV. R9W5 had the lowest ζ-potential but had the highest amount of AMP attached to the hydrogel. This could, again, potentially be explained by the repeating hydrophobic domains of the hydrogel substrate, formed by the Pluronic F-127, and that the more hydrophobic R9W5 prefers this environment compared to the outer surface of the hydrogel. The charge would therefore not be as available on the surface to affect the ζ-potential as much as for R9W4. All the measured surface ζ-potentials are relatively small, and it would therefore be of interest to study how it varies over a larger pH range. However, for the pH-controlled streaming potential measurements, the pH was observed to highly fluctuate upon the acid/base titration steps, most likely due to the high liquid content of the hydrogels which might have buffered the streaming electrolyte solution. To ensure reproducibility, measurements were conducted in a 1:10 dilution of PBS in milli-Q water at pH 7.4, aligning with the electrokinetic analyzer’s maximum ionic strength capacity. This approach balanced physiological relevance and instrument requirements, enhancing the reliability of the obtained values.

The contact angle, as detailed in Table 2, remained largely unchanged, with values ranging between 86–99°, regardless of the AMP used to functionalize the hydrogel. This observation is intriguing as the control hydrogel, despite its propensity to significantly swell in water, exhibited a surprisingly high contact angle of 95.5°. The slightly lower and higher contact angles observed could be explained by the amphiphilic properties of the AMPs. They could theoretically act either as a surfactant, lowering surface tension, or as a hydrophobic coating, all depending on the attachment orientation and hydrophobicity.

From the MIC values, it was clear that when free in solution, changing the cationic amino acid from arginine to lysin resulted in a lower antibacterial effect. For KR9W4 where two arginine were changed to lysin, the observed values were equal to R9W4 for *S. aureus* and 2–4 times higher for *P. aeruginosa*. For K9W4 where all arginine was changed to lysin, the observed MIC was 4–8 times higher for both bacterial strains. This is in line with earlier observations from other studies that also show that lysine-containing AMPs tend to have a lower antibacterial effect compared to arginine-containing AMPs [27,28]. On another note, the theory also suggests that AMPs with a higher hydrophobicity should also result in a stronger antibacterial effect, since a higher hydrophobicity should also increase the affinity towards surfaces, including membranes. However, at first glance, the more hydrophobic version, R9W5, has 2–4 times higher MIC values compared to R9W4, which is the opposite. One explanation could be that during the experiment when the R9W5 was dissolved in the MH-broth, it immediately started to precipitate out of solution. This likely resulted in the actual concentration of free AMP in the solution being lower than the reported value. There are; however, studies that have reported that there might be an optimal level for the antibacterial activity in regards to hydrophobicity as reported by Chen et al. [31]. They observed that the antibacterial activity for a small AMP first increased with the substitution to more hydrophobic amino acids up to a certain point followed by a decrease in antibacterial activity above this point. The hemolytic activity on the other hand only increased with the increase in hydrophobicity, in line with the observations in this present study, at least for the free AMPs.

The antibacterial activity of the hydrogels functionalized with the AMPs against *S. aureus* followed a quite clear trend with respect to the arginine/lysin composition and the tryptophan addition. For hydrogels functionalized with K9W4, where all the arginine was changed to lysin, it had the lowest antibacterial effect of all AMPs with roughly 1 log reduction compared to the control. For hydrogels functionalized with KR9W4, where the two arginine closest to the N-terminal had been changed to lysin, the antibacterial activity against *S. aureus* was almost 10 times higher than hydrogels functionalized with K9W4, but almost 10 times lower than hydrogels functionalized with R9W4. These three AMPs, when attached showed antimicrobial effect against *S. aureus* in a similar trend to that of the MIC when the AMPs were free in solution. The addition of 1 tryptophan in R9W5 slightly increased the antibacterial activity of the AMP functionalized hydrogels compared to R9W4. Against *P. aeruginosa*, the CFU observed was in many cases quite different compared to *S. aureus*. The hydrogels functionalized with the lysin-containing peptides, K9W4 and KR9W4, showed the strongest antibacterial activity. Hydrogels functionalized with R9W4 showed a slightly lower antibacterial activity and hydrogels functionalized with R9W5 even less so.

In contrast to the above, the results from the AMPs attached to the hydrogels against *P. aeruginosa* deviated from the corresponding MIC values with no clear trend. For example, the strong antibacterial effect observed by K9W4 and KR9W4 against *P. aeruginosa* on the hydrogel surfaces is an interesting finding. The notion that end-tagged AMPs behave differently when attached to a surface as opposed to being free in a solution is not implausible. However, the observation where the lysin containing K9W4 and KR9W4 showed a less antibacterial effect in all other tests (i.e., MIC against both strains and against Gram-positive when attached to the surface) compared to the arginine containing R9W4 and R9W5 except against *P. aeruginosa* when attached to the surface lacks a clear explanation. This is a point that warrants further investigation.

Piscidin 1 clearly possesses the strongest antibacterial property when free in solution as very low MIC values of 1–2 µm were observed against *S. aureus* and 8–16 µm against *P. aeruginosa*. Omiganan also showed low MIC values against *S. aureus* with observed concentrations at 4–8 µm, with a bit higher MIC observed against *P. aeruginosa* at 32 µm. Omiganan had quite a low antibacterial effect when present on the hydrogel surface. Upon comparing the Omiganan’s MIC values, as well as the amount of AMP attached to the hydrogel, it has a CFU per hydrogel somewhere between hydrogels functionalized with K9W4 and KR9W4 at roughly 1.5 log reduction compared to the control. Piscidin 1 functionalized hydrogels showed the strongest antibacterial effect against *S. aureus* with more than a 3-log reduction compared to the control. On the other hand, hydrogels functionalized with Piscidin 1 showed quite low antibacterial activity towards *P. aeruginosa.* While still showing a 2-log reduction compared to the control, the antibacterial activity was lower than that of hydrogels functionalized with K9W4, KR9W4, and R9W4. Omiganan functionalized hydrogels showed the lowest antibacterial activity against *P. aeruginosa* with less than 1 log reduction compared to the control. On another note, the observed MIC values for all AMPs were higher against *P. aeruginosa* compared to *S. aureus* even though they were quite low, especially for Piscidin 1 and R9W4. This is not really surprising since *P. aeruginosa* is known to be a very problematic bacteria to eradicate with resistance features against a wide range of antibacterial compounds [38].

The low activity of Omiganan found against both bacterial strains could be due to the peptide’s suggested mode of action of destabilizing the membrane at a certain threshold concentration and potentially the inability to form any secondary structure. The attachment of AMP to the surface of the hydrogel limits its mobility significantly and if they need to aggregate in the membrane to destabilize it, that function will be significantly hampered when covalently attached to a surface. The possibility for Piscidin 1 forming aggregates like pores, suggested to be its mode of action, should also be reduced significantly when bound to the surface, especially since there is a binding site on a lysin in the middle of the structure. However, the situation appears to be more complex since a high antibacterial activity against *S. aureus* was observed but a relatively low antibacterial activity against *P. aeruginosa*. If the feature of adapting an alpha-helical structure is vital for Piscidin 1 to exert its antibacterial effect, it does indeed appear to maintain this feature when attached to the surface of the hydrogel, this is however only speculative at this stage. Something that could potentially explain the different activity observed is the fact that *S. aureus* is Gram-positive and *P. aeruginosa* is Gram-negative. If the Piscidin 1, when attached to the surface, only manages to destabilize the outer membrane of the Gram-negative bacteria and cannot reach further down into the cell wall, this should lead to lower antibacterial activity and could potentially explain the different effects observed.

For the free AMPs, it was evident that the different peptides have very different hemolytic profiles. For the various end-tagged AMPs, distinct trends were once again noticeable for the free peptides. Substituting arginine to lysin resulted in a lower hemolytic effect as K9W4 showed the lowest hemolysis followed by KR9W4. R9W4 started to show higher hemolysis for the higher concentrations although the hemolysis at corresponding MIC values were below 20%. Adding a tryptophan to the sequence clearly shows the effect on the hemolysis since 8 µm for the AMP R9W5 has higher than 20% hemolysis and 32 µm showed 100% hemolysis.

The AMP with the lowest MIC values, Piscidin 1, also had the strongest hemolytic response. At 4 µm Piscidin 1 showed >20% hemolysis and at the observed MIC values against *P. aeruginosa*, 8–16 µm, hemolysis levels for Piscidin 1 were 60–100%. This clearly demonstrates that the very strong antibacterial effect is also accompanied by a strong hemolysis. Omiganan did cross 20% hemolysis at roughly 32 µm, which was also the highest observed MIC against *P. aeruginosa*, but it did not increase to a significantly higher value at higher concentrations.

To assess if the hemolytic effect of the hydrogels was dependent on concentration, four different volumes of 1 vol% blood were used in the experiments. Since the same size of discs (8 mm Ø) was used, lower volumes gave a higher surface/volume ratio. This did; however, not appear to have a significant impact on the observed hemolysis levels. In some cases, especially evident for the control hydrogel, it instead appeared as if the lower volumes got diluted by the hydrogels, as there was a downward trend with observed absorbances lower than the negative control. However, neither hydrogels functionalized with Piscidin 1 nor R9W5, which showed strong hemolysis when free in solution, showed any substantial hemolysis. This indicates that the hemolytic abilities of the AMPs become significantly reduced when they are attached to a surface. Additionally, if there is hemolysis induced by the AMPs attached to the surface but some sort of saturation of the surfaces occurs, the hemolysis levels should have increased by having more surface per volume. The observed dilution effect does; however, make it more difficult to study this. As the AMPs are attached to the hydrogels, their availability for surrounding cells is reduced. For the antibacterial properties, this is evident by the higher amount of AMPs required to kill the bacteria. For eukaryotic cells, where the attraction is already lower to begin with, this reduced availability could explain why no significant hemolysis was reached. Another possible explanation is that the non-fouling nature of the hydrogel itself also plays a role in interaction with the erythrocytes.

The results obtained in this study do not provide enough information to draw conclusions about whether the orientation or attachment site played a significant role in the antibacterial effect or not. It instead appears as if the choice of amino acid plays a larger role in the antibacterial activity. To further study the role of attachment sites and orientation, it would be of interest to block some of the primary amine groups in order to promote certain binding positions. Because, even if there are primary amine groups at the lysine side groups it cannot be ensured that the binding site is evenly spread out among them. It is possible that K9W4, KR9W4, and Piscidin 1 still only, or mainly, attach to the hydrogel by the primary amine at the N-terminal, and this uncertainty is a limitation of the study. Another limitation of the study was that there was no convenient way of analyzing the attachment of Piscidin 1 to the hydrogel, which hampers some of the analysis of this. Regarding adding a tryptophan group in order to obtain a stronger antibacterial effect, the results suggest that it is not as expected. The higher hydrophobicity mainly accomplished solubility issues, questionable antibacterial activity, and for free AMPs, a much higher hemolysis.

When functionalizing a surface with antimicrobial peptides, it is evident that it is not as simple as taking the peptide with the most potent antibacterial effect in solution and using it to attach to a surface. In this study, we show that even with a worked-out hypothesis and supporting theory, the results may prove challenging to predict. Changing and adding a few amino acids can have a tremendous impact on the antibacterial effect and toxicity of an AMP, and the effect might not be the same when free in solution as when attached to a surface. While the antimicrobial mode of action that the peptides adapt when free in solution certainly appears to influence the activity when attached to a surface, this study does not give enough information to draw any definitive conclusion as to why and how. In order to study this further, a more controlled study is required, most likely in combination with simplified liposome vesicles as studying bacterial interaction with materials in detail can be challenging. Furthermore, all presented activity of the AMPs when attached to the material shown in this study might very well be specific for the material support used here. Changing the soft amphiphilic hydrogel base to a rigid metal should have a great impact on the activity of the studied AMPs and a general activity profile is therefore difficult to predict.

## 4. Materials and Methods

### 4.1. Materials

The different peptides used in the present study were purchased with the C-terminals amidated with a guaranteed purity of ≥90% from Genscript (Piscataway, NJ, USA). The peptides were synthesized using Genscript’s PepPower™ technology, which utilizes microwaves in combination with solid-phase synthesis for a high success rate. The polymer, Pluronic F-127 diacrylate, poly(ethylene oxide)_100_-poly(propylene oxide)_70_-poly(ethylene oxide)_100_, with a molecular weight of 12,600 g mol^−1^ and with a guaranteed purity of ≥95% was received as a gift from Amferia AB (Mölndal, Sweden). The EDC (1-ethyl-3-(3-dimethylaminopropyl) carbodiimide), the photoinitiator 2-Hydroxy-4′-(2-hydroxyethoxy)-2-methylpropiophenone, phosphate-buffered saline (PBS), tryptic soy broth (TSB), NaOH, Brain hearth infusion agar (BHI-agar), Muller Hinton (MH) broth, and Triton X-100 were purchased from Sigma Aldrich (Darmstadt, Germany). The N-hydroxysuccinimide (NHS) and MES monohydrate were purchased from Thermo Fisher Scientific (Gothenburg, Sweden).

### 4.2. Preparation of Hydrogels and AMP Attachment

30 wt% Pluronic F-127 diacrylate was first dissolved in water (Milli-Q). The photoinitiator 2-Hydroxy-4′-(2-hydroxyethoxy)-2-methylpropiophenone was then added to the mixture at 0.5 wt% of the Pluronic in the mixture. Everything was mixed until a homogenous white gel was obtained, which in turn was placed at 4 °C until the gel turned into a transparent liquid without any bubbles (usually 2–3 days). The solution was gently poured onto a glass slide, and another glass slide was placed on top with a fixed gap of 1 mm. The construction was left until it reached room temperature, which turned the solution into a viscous gel. It was then placed in a UV curing chamber (UVP Crosslinker; CL-3000 M from Analytik Jena, Jena, Germany, λ = 302 nm) for 90 s per side for a total dose of roughly 0.9 J cm^−2^. For the antibacterial test, spherical discs were punched out with a size of 12 mm Ø from the cross-linked hydrogel sheets. The discs were then washed in plenty of Milli-Q water for at least two days to ensure they were fully swollen (~14 mm Ø) and to remove any non-cross-linked polymer and residual photoinitiator, to prevent interference with AMP attachment or analysis. For the hemolysis test, the whole sheets were washed in water for 3 days and then 8 mm Ø discs were punched out from the swollen and washed sheets.

For the AMP attachment, hydrogel discs were placed in separate wells of a 12-well plate for the 14 mm discs and 48 well plates for the 8 mm discs. To each well, 2 mL (for 14 mm discs) or 0.75 mL (for 8 mm discs) of activation solution (1 mg/mL EDC and 1 mg/mL NHS in 0.5 M MES buffer pH adjusted to 5.5) was added and left for 30 min. The solution was then aspirated, and the hydrogels were washed thrice using Milli-Q water. Then, 2 mL (for 14 mm discs) or 0.75 mL (for 8 mm discs) of 200 µm of each AMP (in PBS) were added to each well in triplicates which were left to react for 2 h. For the controls, only PBS was added. After 2 h, the solutions were aspirated and saved to determine the amount of AMP attachment. The hydrogels were washed thrice using milli-Q water and were then ready to be used for further assessments.

For surface zeta (ζ) potential measurements, the whole sheets were washed in water and manually cut into larger pieces at 2 × 4 cm, followed by swelling in water for a minimum of 3 days. These were placed in individual Petri dishes and activated in 10 mL of activation solution for 30 min and washed with water. This was followed by the addition of 10 mL of 200 µm of the AMP solutions for a 2 h activation. The hydrogels were washed thrice using milli-Q water and finally equilibrated in 1:10 dilution of PBS in milli-Q water for a minimum of 3 h prior to the ζ-potential measurements.

### 4.3. Material Characterization

The AMP attachment was evaluated by saving stock solutions for each AMP and comparing the concentration of the AMP before and after the functionalization. This was performed by UV-vis analysis using a Multiskan GO from Thermo Scientific at 280 nm (absorption of tryptophan). The background of the control hydrogels was subtracted from the measurements and the concentration was then determined by using calibration curves prepared for each AMP.

ζ-potential of the hydrogel surfaces, with and without AMP, was determined by streaming potential measurements using an electrokinetic analyzer (SurPASS 3, Anton Paar GmbH, Graz, Austria) with an asymmetric clamping cell [39]. The hydrogels were prepared as described in the previous section. The electrolyte used for the streaming potential measurements was a 1:10 dilution of PBS in Milli-Q water at pH 7.4 to simulate a physiological pH. The clamping cell containing the hydrogel sample was subjected to four cycles of electrolyte flushing before each new measurement, followed by the recording of four consecutive streaming potential measurements. The fresh electrolyte was introduced for each different hydrogel sample. The ζ-potential values were calculated according to the Helmholtz–Smoluchowski Equation (1) using the measured streaming potential:

(1) $$\zeta =\frac{{dU}_{str}}{d\Delta p}\times \frac{\eta }{\varepsilon_{r}\times \varepsilon_{0}}\times \kappa_{B}$$

where *dU_str_/d*Δ*p* is the streaming potential coefficient (the slope of the streaming potential’s dependence on applied pressure difference), *η* and *ε_r_* × *ε*_0_ are the viscosity and dielectric coefficient of the electrolyte solution (for dilute aqueous solutions coefficient of water are used), and *κ_B_* is the conductivity of the bulk electrolyte solution [40,41]. The calculated ζ-potential values were averaged over four measurements.

The contact angle of the prepared hydrogel discs was evaluated with a Theta Lite optical goniometer from Attension. The hydrogels were placed on the sample holder and a drop of distilled water was placed on top of the hydrogels with the camera recording, the software (OneAttension, version 2.6.0.0) then calculated the contact angle between the material and the surface over the first 10 s of contact time.

### 4.4. Minimum Inhibitory Concentration

The minimum inhibitory concentration protocol was adapted from [42], with the main change in volume for easier observation. In short, the protocol was as follows: a single colony of *Staphylococcus aureus* (CCUG 10778) or *Pseudomonas aeruginosa* (CCUG 56489) was inoculated in 4 mL of TSB and cultivated at 37 °C until OD ~ 0.7 which corresponds to a concentration of roughly 10^9^ CFU mL^−1^. This was then diluted 1000 times to a concentration of roughly 10^6^ CFU mL^−1^ using Mueller Hinton broth (MH broth). In the meantime, the different peptides were weighed in separate 15 mL falcon tubes and dissolved in MH broth for a concentration of 512 µm. Serial dilution by a factor of 2 was then performed, with MH broth, in a 24-well plate, leaving 500 µL of solution in each well. Then, 500 µL of the bacterial solution was added to each well resulting in a starting concentration of 5 × 10^5^ CFU mL^−1^. For the AMP R9W5, the peptide was prepared at a starting concentration of 128 µm due to limited solubility and the highest dilutions were therefore not included. A negative control (no AMPs) and a blank (just MH broth) were included on each plate to confirm that the observed effect was due to AMPs and not anything else, like contamination. The plates were cultivated overnight (16–18 h) and the minimum inhibitory concentration was determined for the lowest concentration where no visible growth was observed. Three separate experiments were performed for all peptides and bacteria.

### 4.5. Antibacterial Evaluation of AMP-Hydrogels

**Figure 3:**
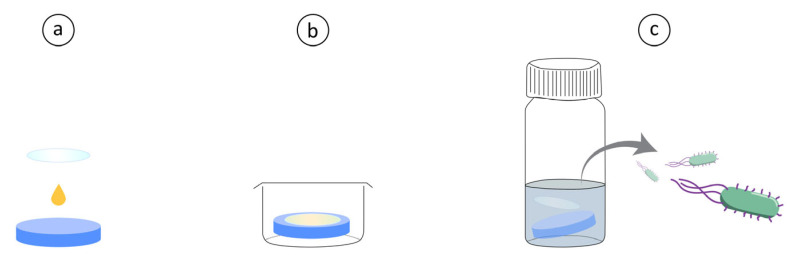
(**a**) A drop of concentrated bacterial solution is placed on top of Hydrogel with or without AMPs. A glass cover slip is placed on top for even distribution. (**b**) Hydrogels with bacteria are incubated in well plates at room temperature for 30 min. (**c**) Hydrogels are then placed in glass vials with 5 mL PBS, vortexed and sonicated. Followed by CFU determination of the PBS solution.

The antibacterial evaluation was inspired by the Japanese Industrial Standard JIS Z 2801 but modified for a shorter contact time utilizing a higher starting concentration, performed as follows. Hydrogel discs were prepared as mentioned above, and a bacterial colony (*S. aureus* or *P. aeruginosa*) was inoculated in TSB to reach mid-log growth (OD ~ 0.7 corresponding to roughly 10^9^ CFU mL^−1^) around the same time as the discs were ready. The bacterial solution was placed in Eppendorf tubes and centrifuged at 5000× *g* for 5 min to form a pellet, the TSB was then removed, and the pellet was resuspended in PBS. Then, 10 µL of the bacterial solution was placed on top of each hydrogel disc and a 13 mm Ø glass cover slip (sterilized by washing with 70% ethanol) was gently placed on top to evenly spread the solution on top of the discs. These were left for 30 min at room temperature after which they were transferred to 4 mL of PBS in separate glass vials using tweezers. Afterwards, 1 mL of PBS was added to each well, washed and then placed in the corresponding glass vial to ensure that all bacteria was collected. The glass vials were vortexed for 10 s, sonicated in an ultrasonic bath for 5 min and then again vortexed for 5 s. The solutions were then diluted and plated by spreading 100 µL of the different dilutions on brain heart infusion agar which were incubated overnight. The plates with colonies in the range 30–300 were then counted, and the original bacterial concentration was calculated. A schematic of the method is shown in Figure 3 for easier visualization. Statistical analysis was performed by first performing a Shapiro–Wilk test on the log_10_ transformed data to ensure a standard distribution and then a *t*-test on the log_10_ transformed data.

### 4.6. Hemolysis

The use of hemolysis assays to study AMPs is a common implementation and quite standardized with small variations [19,43,44]. Serial dilutions of the free AMPs were prepared in PBS to obtain 100 µL in Eppendorf tubes of the concentrations: 2, 4, 8, 16, 32, 64, 128, and 256 µm. A total of 100 µL of just PBS was added to an Eppendorf tube to serve as a negative control, and 100 µL of 0.2% Triton X-100 was added to an Eppendorf tube to serve as a positive control. The functionalized 8 mm Ø hydrogels as well as non-functionalized control hydrogels were placed in Eppendorf tubes together with 0.1, 0.2, 0.3 mL, or 0.4 mL PBS, two samples per type. For each volume, a set of negative control (PBS) and positive control (0.2% Triton X-100) was included using a matching volume. The blood used for the study was defibrinated horse blood from Thermo Fisher Scientific. The blood was placed in a 15 mL falcon tube and centrifuged at 4 °C for 10 min at 1000× *g*. The supernatant was discarded, and the erythrocytes were resuspended in 4 °C PBS and again centrifuged and the supernatant was removed. This washing procedure was repeated until the supernatant was clear, usually 4–5 times. After the final supernatant was removed, 400 µL of the centrifuged erythrocytes were added to 19.6 mL of PBS for a 2 vol% solution. A total of 100 µL of this solution was added to all the samples containing free AMPs, resulting in a final blood concentration of 1 vol% and final AMP concentrations of 1, 2, 4, 8, 16, 32, 64, and 128 µm. For the samples with the hydrogels, the blood solution was added in the same volume as there was PBS in the samples, for a final blood concentration of 1 vol% and total volume of 0.2, 0.4, 0.6, and 0.8 mL. 

The Eppendorf tubes were placed in an incubator at 37 °C for 1 h and the tubes were rotated 3 times every 15 min to limit sedimentation of the red blood cells. The hydrogels were then removed from the tubes using a tweezer and all Eppendorf tubes were centrifuged for 10 min at 1000× *g*. A total of 100 µL of the supernatant from each tube was carefully transferred by pipetting to a 96-well plate. The absorbance was read at 540 nm using a Multiskan GO from Thermo Fisher Scientific. The rate of hemolysis was determined by Equation (2). Three independent runs of the set up were performed. A threshold for hemolysis was set for 20% and a one-sample *t*-test was used to determine the concentration at which this level is crossed.

(2) $$\frac{A_{sample}-A_{negative\;control}}{A_{positive\;control}-A_{negative\;control}}$$

## 5. Conclusions

In this study, we present information about antibacterial and hemolytic activity for six AMPs attached to a soft amphiphilic hydrogel and compare it to the AMPs when free in solution. Changing the amino acid arginine to lysine in a small synthetic AMP with the amino acid sequence RRRPPRPRPRWWWW did not increase the amount of peptide attached, as predicted. However, a significantly lower antibacterial effect was observed for these AMPs when free in solution. When attached to the material, replacing the arginine with lysine gave the AMPs a much lower antibacterial effect against *S. aureus*, but interestingly, slightly higher activity against *P. aeruginosa*. The hemolysis was also lower for the lysine-containing AMP. Adding an extra tryptophan to the sequence resulted in an AMP with much higher hemolytic potential without any obvious antibacterial benefit. In most cases, there was instead a lower antibacterial activity observed, most likely due to an inherent insolubility. Omiganan had a strong antibacterial effect as well as a very low hemolytic effect when free in solution, but the antibacterial effect was almost completely lost when attached to the hydrogel. Piscidin 1 has a strong antibacterial effect free in solution but also a high hemolysis. When attached to the hydrogel, the hemolytic potential was significantly reduced, and a strong antibacterial effect was maintained against *S. aureus,* but a lower antibacterial effect was observed against *P. aeruginosa*.

## Figures and Tables

**Table 1:** Structural information about the peptides used in the study.

Name	Amino Acid Sequence	Number of Amino Acids	Net Charge at pH 7	Hydrophobicity, Wimly White [kcal mol^−1^]
K9W4	KKPKPKPKPWWWW-NH_2_	13	+6	0.65
KR9W4	KKPRPRPRPWWWW-NH_2_	13	+6	1.19
R9W4	RRPRPRPRPWWWW-NH_2_	13	+6	1.55
R9W5	RRPRPRPRPWWWWW-NH_2_	14	+6	3.40
Omiganan	ILRWPWWPWRRK-NH_2_	12	+5	3.95
Piscidin 1	FFHHIFRGIVHVGKTIHRLVTG -NH_2_	22	+4 (+8 at pH < 6)	1.07

**Table 2:** The amount of AMP attached to a 14 Ø mm hydrogel disc ± the standard deviation. *n* = 18. * The uptake of Piscidin 1 could not be determined due to the absence of detectable absorbance in the UV-vis region. The surface ζ-potential at pH 7.4 for the different surfaces, an average of four measurements ± the standard deviation. The water contact angle measured on the different surfaces.

AMP	Attached AMP per Hydrogel Disc (14 mm Ø)	ζ-Potential of Hydrogel Surfaces (at pH 7.4)	Contact Angle
Control	N/A	−1.09 ± 0.08 mV	95.5°
K9W4	119 ± 14 nmol	0.80 ± 0.07 mV	92.4°
KR9W4	136 ± 19 nmol	0.78 ± 0.08 mV	99.6°
R9W4	130 ± 23 nmol	1.41 ± 0.06 mV	86.7°
R9W5	166 ± 28 nmol	0.09 ± 0.09 mV	89.2°
Omiganan	147 ± 26 nmol	0.68 ± 0.04 mV	97.2°
Piscidin 1	N/A *	0.59 ± 0.11 mV	91.8°

**Table 3:** MIC values for the different peptides against *S. aureus* and *P. aeruginosa*. Three independent sets of the measurements were conducted, and if different MIC values were reported for the different runs, a span is presented in the table.

AMP	MIC, *S. aureus* [µm]	MIC, *P. aeruginosa* [µm]
K9W4	32–64	64–128
KR9W4	8–16	16–64
R9W4	8	16
R9W5	16	32–64
Omiganan	4–8	32
Piscidin 1	1–2	8–16

## Data Availability

The data presented in this study are available on request from the corresponding author.
